# Decreased C3 Activation by the *devR* Gene-Disrupted *Mycobacterium tuberculosis* Strain in Comparison to the Wild-Type Strain

**DOI:** 10.1155/2013/512481

**Published:** 2013-05-18

**Authors:** V. Narayan Rao, S. Manivannan, J. S. Tyagi, V. D. Ramanathan

**Affiliations:** ^1^Department of Clinical Pathology, National Institute for Research in Tuberculosis (Formerly Tuberculosis Research Centre) (Indian Council of Medical Research), Mayor V. R. Ramanathan Road, Chetpet, Chennai, Tamil Nadu 600 031, India; ^2^Department of Biotechnology, All India Institute of Medical Sciences, Ansari Nagar, New Delhi 110 029, India

## Abstract

Activation of the complement component C3 is an important step in the complement cascade, contributing to inflammatory mechanisms. Considerable research on gene-disrupted mycobacterial strains using animal models of tuberculosis infection has reported the roles of some of the mycobacterial genes during tuberculosis infection. The aim of the present study was to assess the pattern of complement activation by the *devR* gene-disrupted *Mycobacterium tuberculosis* H37Rv strain and compare with that by its wild-type strain. *In vitro* complement activation at the level of C3 by the gene-disrupted strain, its complemented strain, and wild-type strain was performed using solid-phase ELISA. It was observed that the ability of *devR* gene-disrupted *M. tuberculosis* H37Rv to activate C3 was significantly reduced in comparison to its wild-type strain (*P* < 0.05). In addition, C3 activation by the complemented *devR* mutant strain was almost similar to that of the wild strain, which indicated that the reduced ability to activate C3 could potentially be due to the deletion of *devR* gene. These findings indicate that the gene *devR* probably aids in complement activation and contributes to the inflammatory processes during tuberculosis infection.

## 1. Introduction

The complement system, comprising more than 30 proteins, represents one of the early components that interact with the pathogen. It contributes to host defense against infection directly by its opsonic, inflammatory, and lytic activities and indirectly by enhancing antibody responses. Complement interacts with mycobacteria during the early stages of infection, during which the bacilli activate the complement system and initiate phagocytosis through C3b opsonisation. Several studies are available that report the activation of complement system by many mycobacterial strains [1–4].

Genetic studies since the past few decades have resulted in a number of genetically modified mycobacterial strains, such as the gene-disrupted or mutant strains [5]. Studies on gene-disrupted strains are very useful in delineating the potential roles of the mycobacterial genes during the process of tuberculosis infection [6, 7]. In this context, various mutant strains were studied in animal models of tuberculosis infection for their pathogenicity and virulence properties [8–10]. However, there is lack of information on the complement activation pattern by these gene-disrupted *Mycobacterium tuberculosis* strains. 

Two-component systems play a central role in bacterial adaptation by regulating a spectrum of physiological processes ranging from nutrient uptake to virulence. DevR-DevS (also called as DosR-DosS) is the best characterized two-component system of *M. tuberculosis.* DevR is implicated in the adaptation of *M. tuberculosis* to potential host-derived signals such as hypoxia, nitric oxide, carbon monoxide, or ascorbic acid [11–14] and also in *M. tuberculosis* virulence [9, 15, 16].

Thus, the *devR* gene plays an important role in the survival of *M. tuberculosis* by altering its metabolism during conditions like hypoxia within the host environment. This was also shown *in vivo* by Malhotra et al. [9] who reported that guinea pigs infected with the *devR* gene-disrupted *M. tuberculosis* mutant strain showed a significant decrease in gross lesions in lung, liver, and spleen and only mild pathological changes in liver and lung compared to guinea pigs infected with the parental strain. These findings indicate that the mutant strain was severely attenuated in guinea pigs, which indirectly suggest that the *devR* gene is essential for the survival of *M. tuberculosis* in the host tissues, especially the lung, where harsh conditions like hypoxia prevail during severe tuberculosis infection [9].

Based on the above-mentioned findings, we planned to investigate the interaction pattern of the *devR* gene-disrupted *M. tuberculosis* strain with the human complement system, in particular, the activation pattern of complement by the mutant strain, since there are no studies yet that have looked at this aspect. So, in the present study, *in vitro* complement activation, at the level of C3, by the *devR* gene-disrupted *M. tuberculosis* H3Rv strain and its complemented strain, was studied and compared with that of the parental H3Rv strain. C3 activation was selected, since it is an important complement component where all the pathways of complement activation merge and then proceed to form the membrane attack complex.

## 2. Materials and Methods

### 2.1. Culture of Mycobacteria

The mycobacterial strains—wild-type *Mycobacterium tuberculosis *H37Rv, *devR* gene-disrupted strain, and complemented DevR strain (*devR* gene-disrupted *M. tuberculosis* H37Rv strain complemented with the *devR* gene)—were kindly provided by Dr. Jaya Sivaswami Tyagi, Department of Biotechnology, All India Institute of Medical Sciences (AIIMS), New Delhi, India. The *devR* mutant and complemented mycobacterial strains were constructed at the Department of Biotechnology (AIIMS) [9]. These strains were grown in Middlebrook 7H9 broth media supplemented with 10% albumin dextrose complex (ADC) (BD Biosciences, Franklin Lakes, NJ, USA) and 0.05% Tween-80. Antibiotics, hygromycin (50 *μ*g/mL), kanamycin (25 *μ*g/mL), and cyclohexamide (50 *μ*g/mL) (Sigma Chemical Company, St. Louis, MO, USA),were added as required. The mycobacterial cultures were maintained at 37°C till log phase (3-4 weeks) and then terminated and washed in phosphate-buffered saline (PBS). Heat-killed (at 80°C for 20 min) bacilli were counted using a Thoma counting chamber and aliquoted into small vials in appropriate numbers, for use in C3 activation experiments.

### 2.2. Source of Complement

Pooled normal human serum (NHS) obtained from 10 healthy laboratory volunteers was used as the source of complement for C3 activation studies. The drawing of the blood from the volunteers, for sera preparation, was done by a trained technician under medical supervision. The sera were aliquoted into microcentrifuge vials and stored at −80°C, which were used only once after thawing. For demonstration of complement classical pathway, the sera were diluted in phosphate-buffered saline (PBS) tween 20 (PBST) containing Ca^++^ and Mg^++^ ions; for alternative pathway, PBST containing MgEGTA was used to dilute the serum. Serum diluted in PBST-EDTA was used as control, which blocks complement activation.

### 2.3. Enzyme-Linked Immunosorbent Assay

Solid-phase enzyme-linked immunosorbent assay (ELISA) was performed using the above mycobacterial strains to assess complement activation at the level of C3. Briefly, a 96-well immunoassay ELISA plate was coated with the heat-killed mycobacterial strains (100 *μ*L/well) in 10^8^/mL or 10^7^/mL or 10^6^/mL numbers and incubated overnight at 4°C. Next day, the wells were emptied and tapped on an absorbent paper and 100 *μ*L of 3% bovine serum albumin (BSA) was added to the wells to block the excess antigen binding sites. Then, the plate was incubated at 37°C for 1 hour, after which 50 *μ*L of NHS diluted in various buffers, namely, PBST-Ca^++^Mg^++^ or PBST-MgEGTA or PBST-EDTA was added to the wells and incubated. NHS diluted in PBST-EDTA served as the control for the ELISA experiments, since EDTA blocks complement activation. Then the plate was washed three times using PBST, and 50 *μ*L of antihuman C3 antibody raised in rabbit, conjugated with HRP (DAKO, Denmark), was added to the wells and incubated as above. After washing the plate with PBST, 50 *μ*L of tetra methyl benzidine was added and incubated for 15 min at room temperature in the dark. Washing was repeated, and the reaction was stopped using 25 *μ*L of 0.5 M H_2_SO_4_. Finally, the plate was read for optical density (OD) values in an ELISA Reader (Molecular Devices, Sunnyvale, CA, USA) at 450 nm.

## 3. Statistical Analysis

SPSS (version 16.0) was used to analyse the statistical differences between complement activation pattern by the mutant strains and that of the wild-type H37Rv strain. Each ELISA experiment was performed three times with the mutant and the wild-type strains in triplicate wells in the immunoassay plate. The mean values obtained in these experiments were used for the statistical analysis, and the results are expressed as average OD values ± SEM. Student's *t*-test was used for statistical analyses and *P* values less than 0.05 were considered as statistically significant.

Ethical clearance was obtained from the Institutional Ethics Committee to perform the study. The laboratory volunteers were given a detailed explanation of the study and informed consent was obtained for drawing blood samples for preparing the NHS.

## 4. Results

C3 activation by the *devR *gene-disrupted strain, its complemented strain, and parental *M. tuberculosis* H37Rv strains was assessed through both classical and alternative pathways using ELISA. NHS dilutions of 1/100 for classical pathway and 1/10 for the alternative pathway were used for the experiments.

**Figure 1:**
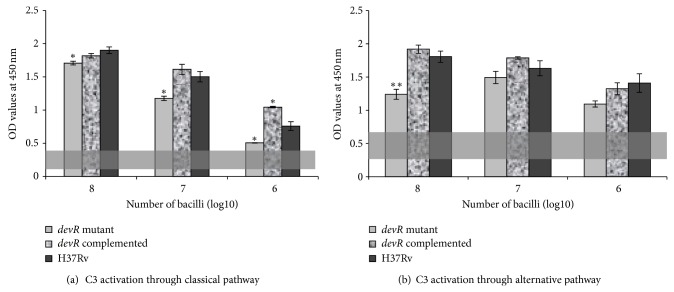
Pattern of C3 activation by the different mycobacterial strains. (a) C3 activation through classical pathway by *devR* mutant, complemented, and wild-type *M. tuberculosis* H37Rv mycobacterial strains. (b) C3 activation through alternative pathway by *devR* mutant, complemented, and wild-type *M. tuberculosis* H37Rv mycobacterial strains. ^*^ *P* < 0.05; ^**^ *P* < 0.01. The shaded bars represent limits of C3 activation by blocking complement activation using normal human serum diluted in PBST-EDTA.

Figure 1 shows the C3 activation profile by the various mycobacterial strains used in this study. The mutant strain exhibited statistically significantly decreased C3 activation through both the classical and alternative pathways. C3 activation was decreased by >20% (*P* = 0.02) and >30% (*P* = 0.03) by the mutant strain at 10^7^/mL and 10^6^/mL bacilli numbers, respectively, compared to that by the wild-type parental H37Rv strain. At 10^8^/mL, the decrease was about 10%, which was also significant (*P* = 0.03) (Figure 1(a)). C3 activation was also reduced through the alternative pathway by the *devR* mutant strain, although not statistically significant at 10^7^/mL and 10^6^/mL bacilli numbers. Only at 10^8^/mL of bacilli numbers, there was >30% reduction in C3 activation, which was statistically significant compared to that by the wild-type strain (*P* < 0.01) (Figure 1(b)).

By contrast, the complemented strain exhibited increased levels of C3 activation at all bacilli numbers, compared to that by the mutant strain. In fact, C3 activation by the complemented DevR strain was almost equal to that of the wild-type parental *M. tuberculosis* strain through both the activation pathways, with no significant difference (Figure 1). However, only at 10^6^/mL of the complemented strain, C3 activation was significantly more than that by the wild-type strain (*P* < 0.05) through the classical pathway (Figure 1(a)). These findings are interesting, since, theoretically, the complemented strain would revert the changes whatsoever caused by the mutant strain. Hence, the increase in complement activation by the complemented strain to almost similar levels to those by the wild-type strain stresses the fact that deletion of *devR* gene results in decrease of complement activation as observed for the mutant strain.

## 5. Discussion

The complement system with the various proteins and receptors interacts with the bacterium leading to opsonisation and phagocytosis. Ferguson et al. [4] had reported that the complement system likely plays a role in the pathogenesis of tuberculosis during the innate immune response by opsonising *M. tuberculosis* with specific C3 cleavage products for phagocytosis into the alveolar macrophage through activation of the classical complement pathway in the lung. In addition, prior studies have established that BCG activates the alternative complement pathway and that the virulent Erdman strain of *M. tuberculosis* activates complement and fixes C3 protein [1, 17].

It is well known since decades that infection with mycobacteria results in the formation of an inflammatory granuloma. The possible role of complement in the production and the maintenance of granuloma was earlier suggested by Schorlemmer et al. [18]. These authors found that a variety of agents that induce chronic granulomatous inflammation were able to activate complement and also to release enzymes from macrophages. This results in inflammation, thereby inducing the macrophages to release lysosomal enzymes and complement components [18]. The released lysosomal enzymes activate complement, which, in turn, become haemotactic to macrophages and also activate them [19]. These events could then lead to the formation of a granuloma.

In the present investigation, the *devR* gene-disrupted *M. tuberculosis* H37Rv strain exhibited reduced potential to activate C3, which could imply that deletion of the *devR* gene has modified the properties of the bacillus in its interaction with complement system. Such an observation implies that, probably, the gene *devR* aids C3 activation during the infectious process, and this could be one mechanism by which *M. tuberculosis* not only gains entry into macrophages but also contributes to the formation of a chronic granuloma, as earlier suggested by Schorlemmer et al. [18]. In addition, Malhotra et al. [9] reported that the *devR* mutant strain was attenuated in virulence, as depicted by fewer gross lesions and organ pathology and a nearly 1000-fold lower bacterial load in guinea pigs infected with the *devR* mutant strain compared to guinea pigs infected with the parental strain. They also observed that the mutant strain failed to cause severe progressive disease and pathology as compared to the wild-type strain, under their experimental conditions. Decreased C3 activation by the mutant strain as observed in the present investigation further stresses the significance of *devR* gene in the inflammation and pathogenesis of tuberculosis.

The study has few limitations. Using gene-disrupted strains in mycobacterial research has both advantages and a few drawbacks. Each gene contributes to some extent for the survival of the bacillus; however, *M. tuberculosis* has adapted to a more obstinate behaviour in the human host so that the functional loss of a particular gene might be compensated by another to maintain survival. Therefore, the functional properties of a particular gene cannot be elucidated precisely. Theoretically speaking, the deletion of a group of genes, rather than one particular gene, could offer more insights into the metabolic and physiological aspects of *M. tuberculosis* and the functions of the gene(s) could be studied in more detail. Moreover, since the study is only a preliminary analysis of assessing the activation pattern of complement at the level of C3 by the mutant strain, further experiments, such as macrophage phagocytosis and molecular studies to identify which translated component of the *devR* gene was responsible for complement activation, were not performed to arrive at a definite conclusion. This is another limitation of the present study. However, the present findings do provide information that deletion of the *devR* gene from the genome of *M. tuberculosis* alters the properties of the mycobacterial strain by displaying a reduced ability to activate complement at the level of C3. The *devR* gene complemented *M. tuberculosis* strain displayed almost similar C3 activation levels as that of the wild-type strain, which emphasizes the fact that deletion of the *devR* gene is indeed responsible for the reduced C3 activation.

In the context of pathogenesis, it has been already documented that *devR* gene mutant strain has a reduced ability to survive in the host [9]. Since complement activation significantly contributes to important physiological processes such as inflammation and phagocytosis, reduction in activation of C3 might possibly alter or impair these processes, by which the mycobacteria might get attenuated in the host and display less pathogenicity, as observed by Malhotra et al. [9] in their study using guinea pigs.

In conclusion, the present study showed that the mutant *devR M. tuberculosis* strain has a reduced ability to activate C3 compared to its wild-type strain. However, further studies on the molecular and genetic aspects and using animal models of tuberculosis infection could throw more light on this aspect. Studies such as the present one can be beneficial in providing more knowledge in the context of identifying drug targets in the mycobacterial genome, which could pave way for further research in creating novel drugs against tuberculosis.
